# Vitamin D supports activated CD4^+^ T-cell proliferation through enhanced glutaminolysis

**DOI:** 10.3389/fimmu.2026.1791098

**Published:** 2026-06-11

**Authors:** Agustín A. Vera, Mauricio Hernandez, Ricardo A. Cartes, Faryd Llerena, Solange E. Cisterna, Romina A. Quiroga, Sergio A. Sanhueza, Camila P. Muñoz-Grez, Francisco Vergara, Daniel Moena, Pablo A. Alarcón, Rafael Burgos, Coralia I. Rivas, Elena Uribe, Rafael J. Argüello, Cristopher Almarza, Felix A. Urra, Francisco Tapia-Belmonte, Luciano Ferrada, Liliana I. Lamperti, Estefanía Nova-Lamperti

**Affiliations:** 1 Molecular and Translational Immunology Laboratory, Department of Clinical Biochemistry and Immunology, Pharmacy Faculty, University of Concepcion;, Concepcion, Chile; 2 Division of Biotechnology, MELISA Institute, Concepción, Chile; 3 Facultad de Derecho y Ciencias Sociales, Universidad San Sebastián, Concepción, Chile; 4 Facultad de Odontología, Universidad San Sebastián, Concepción, Chile; 5 Departamento de Química y Medio Ambiente, Universidad Técnica Federico Santa María, Valparaíso, Chile; 6 Institute of Pharmacology and Morphophysiology, Faculty of Veterinary Sciences, Austral University of Chile, Valdivia, Chile; 7 Departamento de Fisiopatología, Facultad de Ciencias Biológicas, Universidad de Concepción, Concepción, Chile; 8 Biochemistry and Molecular Biology Department, Universidad de Concepción, Concepción, Chile; 9 Aix Marseille Univ, CNRS, INSERM, CIML, Centre d’Immunologie de Marseille-Luminy, Marseille, France; 10 Laboratory of Metabolic Plasticity and Bioenergetics, Interdisciplinary Nucleus of Pharmacology & Immunology (ICBM), Medical Faculty, University of Chile, Santiago, Chile; 11 Cancer Molecular Dynamics Laboratory, Pharmacology Department, Faculty of Biological Sciences, Universidad de Concepción, Concepción, Chile; 12 Centro de Microscopía Avanzada (CMA) BIO BIO, Faculty of Biological Sciences, Universidad de Concepción;, Concepción, Chile

**Keywords:** glutaminolysis, immunometabolism, T cell proliferation, T cell regulation, vitamin D

## Abstract

**Background:**

Vitamin D (VitD) is an important immunometabolic regulator of T cell function. Its active form, 1,25-dihydroxyvitamin D_3_, signals through the VitD receptor (VDR), which is highly expressed in activated CD4^+^ T cells. Although VDR signaling suppresses glycolysis by reducing glucose uptake and glycolytic enzyme expression, early T cell expansion is preserved, suggesting the involvement of alternative metabolic pathways. Since glutaminolysis is essential for T cell activation and proliferation, we investigated whether VitD modulates glutamine metabolism during early CD4^+^ T cell activation.

**Methods:**

Human CD4^+^ T cells were stimulated with αCD3/CD28 for four days in the presence or absence of VitD, and analyzed using complementary metabolic, proteomic, and functional approaches.

**Results:**

VitD-treated cultures exhibited increased cell numbers despite reduced glucose uptake and lactate production, indicating proliferation partially independent of classical glycolytic metabolism. Proteomic analysis revealed increased expression of glutaminase, glutamate dehydrogenase, and CD38, together with enrichment of Selenium Metabolism and Selenoproteins and Nicotinate and Nicotinamide Metabolism, suggesting enhanced glutaminolysis and NAD+ remodeling. Consistently, tritiated and ¹³C-glutamine tracing demonstrated increased glutamine uptake and incorporation into glutamate, α-ketoglutarate, glucose, and inositol-related metabolites, supporting a glutaminolysis-dependent anabolic program rather than oxidative phosphorylation. Pharmacological inhibition of VDR (MeTC7, 1 nM), glutamine uptake (GPNA, 250 µM), or glutaminase activity (BPTES and compound 968, 5 µM) significantly reduced T cell expansion, highlighting glutamine metabolism as essential for the VitD-mediated cell expansion. Interestingly, prolonged cultures showed that VitD ultimately restricted proliferation at day 7; however, supplementation with glutamine and VitD restored cell expansion, suggesting that VitD promotes a metabolically restrained but adaptive proliferative state.

**Discussion:**

Overall, our findings identify glutaminolysis as a central metabolic pathway supporting VitD-induced CD4^+^ T cell expansion independently of canonical glycolytic and OXPHOS-associated programs, while promoting metabolic resilience and inflammatory restraint.

## Graphical Abstract

**Figure:**
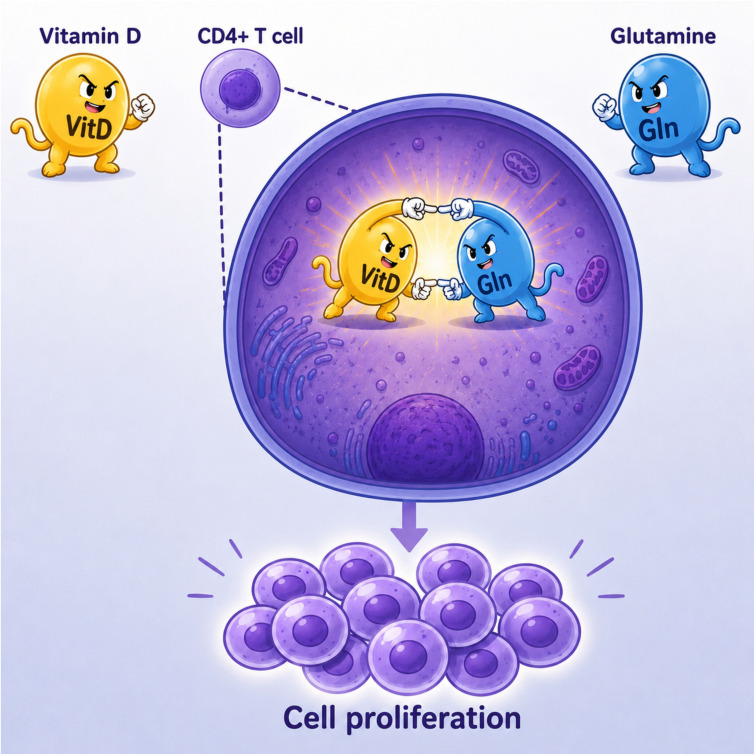

## 1 Introduction

Vitamin D (VitD) plays a central role in immune regulation, as its receptor (VDR) is expressed in most immune cells, including antigen-presenting cells (APCs), and both T and B lymphocytes. Immune cells also express the enzyme Cyp27B1, which catalyzes the second hydroxylation required to generate the active metabolite of VitD (1, 2). Upon T cell activation, VDR expression is upregulated (3, 4), enabling VitD to exert stronger transcriptional and functional effects. Through this pathway, VitD modulates the expression of key transcription factors and cytokines that shape T cell differentiation. For example, VitD induces methylation of the IFN-γ gene, thereby inhibiting Th1 differentiation (5, 6), reduces IL-17 production to limit Th17 responses, and promotes the expression of IL-4, IL-5, IL-10, and IL-13, favoring Th2 and regulatory T cell (Treg) development (7, 8). Together, these mechanisms establish VitD as a critical regulator of adaptive immunity with predominantly anti-inflammatory and pro-resolutive effects.

T cell activation is accompanied by profound metabolic reprogramming to support the bioenergetic and biosynthetic demands of clonal expansion. Activated CD4^+^ T lymphocytes increase glucose uptake up to 20-fold and adopt a highly glycolytic phenotype, converting approximately 85% of glucose to lactate even in the presence of oxygen (9, 10). This aerobic glycolysis, reminiscent of the Warburg effect, enables rapid ATP generation and provides intermediates required for macromolecule synthesis (11). Although glucose is the primary substrate supporting early activation and proliferation, other nutrients also contribute to biomass accumulation, including fatty acids for membrane synthesis and amino acids for protein and anabolic metabolism (12, 13).

Interestingly, when VitD is present during T cell activation, a paradoxical metabolic response emerges. Previous work from our group demonstrated increased cell numbers at early stages post-activation in VitD-treated cultures (7), despite a marked suppression of aerobic glycolysis in activated human CD4^+^ T cells (14). These findings suggest that VitD redirects metabolic flux away from glycolysis, prompting the engagement of alternative pathways to sustain proliferation (13). However, the metabolic substrate compensating for reduced glycolytic output under VitD exposure remains unclear.

Glutamine, the most abundant amino acid in human plasma, represents a strong candidate to fulfill this role. It accounts for 40–60% of circulating amino acids and becomes essential under metabolic stress conditions such as infection or inflammation (15–17). Glutamine is indispensable for immune cell proliferation, differentiation, and survival, particularly in activated CD4^+^ T lymphocytes (16, 17). Its uptake is primarily mediated by the transporters ASCT2 (SLC1A5) and SNAT1 (SLC38A1) (18, 19), which together ensure efficient and sustained glutamine influx across physiological concentration ranges (19–21). Once internalized, glutamine supports energy production, nucleotide and protein synthesis, redox balance, and signaling pathways that regulate immune activation (22). Through glutaminolysis, glutamine is converted to α-ketoglutarate, fueling the tricarboxylic acid (TCA) cycle and supporting oxidative and biosynthetic processes essential for T cell expansion (23).

Given its central role in proliferating cells, glutamine metabolism has been extensively targeted in cancer research. Pharmacological inhibitors such as GPNA, which blocks glutamine uptake via ASCT2, and glutaminase inhibitors including BPTES and Compound 968, impair ATP production and cell proliferation in multiple tumor models (24–26). In T cell biology, these compounds provide valuable tools to interrogate glutamine dependence during activation and differentiation.

Despite extensive characterization of VitD immunoregulatory functions, its impact on glutaminolysis and glutamine transport during T cell activation remains poorly defined. Therefore, this study aims to investigate the role of glutaminolysis in cell number, viability, and glutamine transport in CD4^+^ T lymphocytes activated in the presence of VitD.

## 2 Materials and methods

### 2.1 Isolation of CD4^+^ T lymphocytes

Memory CD4^+^ T cells were isolated from approximately 40 mL of blood obtained from healthy donors recruited under Institutional Ethic Committee (PAI N79170082). Donors provided informed consent, and samples were collected on the same day. Blood samples were diluted with PBS (1:1) and layered on Ficoll (1:1) in 50 mL conical tubes. Mononuclear cells were isolated by density gradient centrifugation at 2000 rpm for 30 min at room temperature. The mononuclear layer was collected, washed with PBS, and centrifuged at 1500 rpm for 10 min. Cells were resuspended in 1 mL of PBS, and the total cell count was performed using trypan blue and a Neubauer chamber. For the isolation of CD4^+^ T cells, the Memory CD4+ T Cell Isolation Kit Human (Miltenyi Biotec, Bergisch Gladbach, Germany) was used. After cell counting, the protocol was adjusted according to the manufacturer’s instructions. Finally, cells were stained with anti-CD4 FITC or anti-CD4 APC-Cy7 (BioLegend, CA, USA) and sorted using a BD FACSAria II cell sorter (BD Biosciences, CA, USA). A fluorescence-minus-one (FMO) control was used to ensure the purity of the sorted cell population.

### 2.2 Culture conditions and viability analysis

Cells were cultured in round-bottom 96-well plates with 200 µL per well, containing 1×10^5^ cells. Cultures were maintained in RPMI 1640 medium (Gibco, MA, USA) supplemented with 2 mM glutamine, either in the presence or absence of 1,25-dihydroxyvitamin D (VitD) (final concentration 10 nM in ethanol, Enzo Life Sciences), the concentration of VitD was selected based on previous studies, including our own, demonstrating reproducible immunomodulatory effects on human CD4^+^ T cells (7). Cells were activated using anti-CD3/CD28 microbeads (6.25 µL per 1 million cells; 1:4 ratio, ThermoFisher Scientific, USA) and incubated at 37°C in 5% CO_2_. Controls without VitD were treated with the same amount of ethanol as carrier. Experiments were carried for 4 to 7 days after initial activation. For glutamine deprivation experiments, culture medium was replaced on day 4 with RPMI 1640 supplemented or not with glutamine, according to experimental conditions. In selected prolonged culture conditions, VitD or Carrier was re-added after medium replacement. For viability and phenotypic analysis, cells were stained with Live/Dead APC dye (ThermoFisher Scientific, USA) (1:1000 dilution), anti-CD38 (BioLegend), anti-CD45RA (BioLegend) and anti-CCR7 (BioLegend) and incubated at 4 °C for 15 min in the dark. After washing with PBS (1500 rpm, 5 min), cells were resuspended in 100 µL of PBS with CountBright™ absolute counting beads (Invitrogen, USA) and analyzed by flow cytometry using LSR-Fortessa X20 (BD Biosciences, CA, USA). For cell proliferation cell were labelled with Cell Trace Violet (1µM, ThermoFisher Scientific, USA). For inhibitor studies, concentration curves were established for GPNA (ASCT2 inhibitor, 100-250 µM, G6133 Merck-SIGMA), inhibitor 968 (glutaminase inhibitors, 5-25 µM, SML1327 Merck-SIGMA), BPTES (glutaminase inhibitors, 5-25 µM, SML0601 Merck-SIGMA) and MeTC7 (VDR antagonist, 1 nM, HY-147337, MedChemExpress). Cells were activated for two days prior inhibitors’ addition for GPNA, 968 and BPTES. Intracellular NAD+/NADH levels were measured using the NAD+/NADH Assay Kit (K337-100, BioVision), following the manufacturer’s instructions.

### 2.3 Immunoblotting

Proteins were extracted by lysing the samples for 30 min at 4 °C under constant agitation in RIPA buffer (Thermo Fisher Scientific, #89901) supplemented with protease and phosphatase inhibitor cocktail (Cell Signaling Technology, #5872). The homogenates were subsequently centrifuged at 12,000 rpm for 15 min at 4 °C, and the supernatants were collected for further analysis. Protein concentration was determined using Bradford method according to the manufacturer’s instructions. For Western blot analysis, 30 µg of total protein were loaded and separated on 10% TGX FastCast acrylamide gels (Bio-Rad, #1610173). Proteins were then transferred onto PVDF membranes (0.45 µm pore size; Immobilon-P, Merck Millipore, #IPVH00010). Membranes were probed with primary antibodies against GLS (Thermo Fisher Scientific, Cat# MA5-49192) (1:1000) and β-actin (Santa Cruz Biotechnology, Cat# sc-47778, HRP-conjugated) (1:10.000), followed by appropriate HRP-conjugated secondary antibodies when required. Immunoreactive bands were detected using an enhanced chemiluminescence (ECL) substrate according to the manufacturer’s instructions.

### 2.4 Measurement of glucose consumption and lactate production

Glucose consumption and lactate production were measured in supernatants collected from CD4^+^ T lymphocyte cultures after 2 and 4-days of culture. Samples were diluted 1:10 with distilled water, and 10 µL of this dilution was added to a 96-well plate. For both, glucose and lactate measurements, a standard curve was prepared using the human calibrator AX125 (BioSystems, Barcelona, Spain). For glucose measurement, 200 µL of the BioSistem Glucose reagent (Cat: 12503; Biosystems, Barcelona, Spain) was added to each well and incubated at room temperature for 10 min. Absorbance was measured at 500 nm using Synergy™ 2 Microplate reader (BioTek Instruments, Bad Friedrichshall, Germany). Lactate production was determined using the BioSystem Lactate Kit (COD 12736; BioSystems, Barcelona, Spain). A mix of 80 µL of reagent A and 20 µL of reagent B was prepared and added to each well. After a 5-min incubation at 37 °C, absorbance was measured at 600 nm using the same Synergy™ 2 Microplate reader (BioTek Instruments, Bad Friedrichshall, Germany). In both cases, data were analyzed using Gen5 software (Agilent, CA, USA), and the resulting concentrations were normalized to cell counts using CountBright™ absolute counting beads (Life Technologies, CA, USA).

### 2.5 Glucose uptake with 2-NBDG

Glucose uptake was assessed using the fluorescent marker 2-(N-(7-nitrobenz-2-oxa-1,3-diazol-4-yl)amino)-2-deoxyglucose (2-NBDG, Invitrogen, USA) following the manufacturer’s instructions. Briefly, cells were rested without glucose form 1 h. Then, cells were collected into flow cytometry tubes and centrifuged at 1500 rpm for 10 min. After removing the supernatant, cells were resuspended in 100 µL of PBS and subsequently mixed with 100 µL of PBS containing 2-NBDG at a concentration of 20 µM, resulting in a final concentration of 10 µM. Cells were incubated at 37 °C for 30 min. The reaction was stopped by adding 800 µL of cold PBS, and cells were centrifuged again at 1500 rpm for 10 min. After centrifugation, cells were stained with Live/Dead dye (ThermoFisher Scientific, USA) and 5 µL of CountBright™ absolute counting beads were added. Samples were then acquired using the LSRFortessaX20 system (BD Biosciences, CA, USA) and analyzed using FlowJo software (FlowJo LLC, OR, USA).

### 2.6 Glutamine transport assay

CD4^+^ T cells were incubated with various concentrations of cold glutamine (0 to 200 µM) and [^3^H] glutamine (5 µCi/mL) for 1 min at 37 °C to assess initial uptakes rates, either in the presence or absence of VitD. After incubation, cells were washed with 800 µL of ice-cold PBS containing 2 mM glutamine. Pellets were lysed using 200 µL of 0.2 N NaOH, followed by agitation for 30 min. The lysate (100 µL) was transferred to scintillation vials with 2.8 mL of scintillation liquid for analysis using the LS 6500 Multi-Purpose Scintillation Counter. Protein quantification was performed on the remaining 100 µL. Glutamine uptake was determined using Michaelis-Menten equations to calculate Vmax and Km (**Supplementary Figure 1**). Variations were expressed as mean ± standard error.

### 2.7 Proteomics of CD4^+^ T cells

2×10^5^ CD4^+^ T cells from healthy donors were activated with anti-CD3/CD28 beads (1:5 ratio) in XVIVO-15 at 37 °C in the presence or absence of VitD (10nM) for 5 days. Cells were washed and stored with protease inhibitor before proteomics analysis. Samples were analyzed using a nanoELUTE (Bruker Daltonics, Bremen, Germany) ultrahigh-pressure nanoflow chromatography system coupled online to a hybrid trapped ion mobility spectrometry-quadrupole-time-of-flight mass spectrometer (timsTOF Pro, Bruker Daltonics, Bremen, Germany) with a modified nanoelectrospray ion source (CaptiveSpray, Bruker Daltonics). Liquid chromatography was performed (50 °C, 400 nL/min constant flow on a reversed-phase column Aurora Series CSI (25 cm × 75µm i.d C18 1.6 µm) (ionopticks Australia)). Mobile phases A and B were watered with 0.1% formic acid (v/v) and 99.9/0.1% ACN/formic acid (v/v), respectively. Tandem mass spectra were extracted by Tims Control version 2.0. Charge state deconvolution and deisotoping were not performed. All MS/MS samples were analyzed using MSFragger 3.2 set up and to search the Uniprot_SwissProt database (unknown version, 21.040 entries) assuming the digestion enzyme trypsin. MSFragger was searched with a fragment ion mass tolerance of 20ppm and a parent ion tolerance of 40ppm. The carbamidomethyl of cysteine was specified in MSFragger as a fixed modification, the quantification used IonQuant for Label Free, and the peptide validation used Peptide Prophet. The quantitative (LQF) and statistical analysis used perseus software version 1.615 (Max Planck Institute, Martinsried, Germany). Detailed analysis was performed as previously published (27).

### 2.8 Bioinformatics analysis

Mass Spectrometry Data Processing: Protein identification and label-free quantification were performed using *MaxQuant* (version 2.7.5.0) (28). Protein abundances were estimated using the MaxLFQ algorithm (29), which computes label-free quantification (LFQ) intensities from peptide-level data. Database searches were conducted against the human UniProt/Swiss-Prot reference proteome. The resulting MaxLFQ intensity values were used as the primary measure of relative protein abundance for all downstream analyses.

### 2.9 Data cleaning and preprocessing

Raw MaxQuant output was processed in Python using the *pandas* and *NumPy* libraries. MaxLFQ intensity columns corresponding to cell lysate samples were extracted from the combined protein groups table. Proteins flagged as potential contaminants (i.e., those whose UniProt accession contained the string “contam”) were removed prior to further analysis. UniProt accessions of the remaining proteins were mapped to official HGNC gene symbols using the BioMart API, accessed through the *biomart* module of the GSEApy library (version 1.2.1) (30). Proteins for which no gene symbol could be retrieved were excluded. The final preprocessed dataset contained the UniProt accession, gene symbol, protein description, and per-sample MaxLFQ intensities for each of the three biological replicates per treatment group.

### 2.10 Differential abundance analysis

Differential protein abundance analysis was performed in R (version 4.6.0) using the *proteoDA* package (version 1.0.1) (31), which provides a structured, pipe-friendly workflow built on the *limma* linear modelling framework (version 3.64.3) (32). The analysis was carried out through the following sequential steps:

- Missing value imputation: intensity values equal to zero were converted to missing values (NA) using the zero_to_missing() function.
- Protein filtering: only proteins with valid quantification in at least 66% of samples within at least one treatment group were retained (min_prop = 0.66, filter_proteins_by_proportion()). The adequacy of this filtering criterion was assessed through a pre-normalization diagnostic report generated by write_norm_report().
- Normalization: filtered intensity values were normalized using quantile normalization (normalize_data(), norm_method = “quantile”).
- Quality control: a comprehensive QC report was generated using write_qc_report(), comprising a principal component analysis (PCA, axes 1 and 2), a hierarchical clustering heatmap based on Euclidean distance with complete linkage, and an assessment of the 500 most variable proteins.
- Statistical model: a no-intercept linear model was fitted on the treatment variable (~0 + treatment) using add_design(). A single contrast was defined comparing the vitamin D-treated condition to the ethanol vehicle control (VitD_vs_Eth = Vitd − Eth) via add_contrasts(), and the model was fitted using fit_limma_model().
- Result extraction: differentially abundant proteins were identified using extract_DA_results() with an adjusted p-value threshold of ≤ 0.05 (Benjamini–Hochberg correction, BH) and a minimum absolute log2 fold-change of 0.5 (|log2FC| ≥ 0.5).

### 2.11 Gene set enrichment analysis (Pre-ranked GSEA)

To explore biologically relevant pathways enriched under VitD treatment, a pre-ranked Gene Set Enrichment Analysis (GSEA) was performed using the *prerank* module of the GSEApy library (version 1.2.1) (30). Proteins were ranked from highest to lowest according to their limma-derived moderated t-statistic, which incorporates both the magnitude and the statistical significance of abundance changes.

The analysis was conducted against eight human gene set databases: KEGG_2026, KEGG_2021_Human, Reactome_Pathways_2024, WikiPathways_2024_Human, HumanCyc_2016, BioPlanet_2019, HMDB_Metabolites, and MSigDB_Hallmark_2020. The following parameters were applied: minimum gene set size of 10 (min_size = 10), maximum gene set size of 500 (max_size = 500), 1,000 permutations (permutation_num = 1000), and a fixed random seed of 42 to ensure reproducibility. Given the exploratory nature of this analysis, gene sets were retained for further inspection at a relaxed FDR q-value threshold of ≤ 0.6. From this broad candidate pool, a curated subset of biologically relevant pathways was selected for visualization based on functional coherence and biological plausibility.

### 2.12 Data visualization

All visualizations were generated in Python using the matplotlib and seaborn libraries. Three complementary figures were produced:

- Volcano plot: differential abundance results were visualized as a scatter plot of log2 fold-change (log2FC) on the x-axis versus −log10 of the adjusted p-value (−log10 adj.P.Val) on the y-axis. Proteins were classified as overexpressed (log2FC > 0.5, adj.P.Val ≤ 0.05), underexpressed (log2FC < −0.5, adj.P.Val ≤ 0.05), or unchanged. Selected proteins of biological interest were annotated using non-overlapping labels positioned automatically with the adjustText library.
- Heatmap: a hierarchical clustering heatmap was generated for a selected panel of proteins of interest using seaborn’s clustermap function. Intensity values were z-score normalized per protein, and a diverging color palette (blue–white–red) was applied. Rows (proteins) were clustered hierarchically while columns (samples) retained their original group order.
- GSEA dotplot: enriched pathways were visualized as a dot plot in which each point represents a gene set. The x-axis displays the Normalized Enrichment Score (NES), the dot size encodes the percentage of leading-edge genes from the input list (Gene %), and the dot color reflects the FDR q-value. Pathways were color-coded by functional category: metabolic pathways (green), apoptosis (red), and AKT/PI3K/AMPK signaling (blue).

### 2.13 Isotopic tracing of glutamine metabolism using [1-¹³C]-L-glutamine

To determine the metabolic fate of glutamine following its uptake by lymphocytes, isotopic tracing experiments were performed using [1-¹³C]-L-glutamine (MedChemExpress, Cat. No.: HY-N0390S5). All experiments were conducted in the laboratory of Dr. Rafael Burgos, Universidad Austral de Chile, following established protocols for immune cell metabolomics (33). Briefly, CD4^+^ T cells were purified and activated at 37 °C 5% CO_2_. Where indicated, cells were treated with VitD at 10 nM. After 96 h of activation, culture medium was replaced with glutamine-free RPMI 1640 supplemented with [1-¹³C]-L-glutamine (1 mM final concentration). Cells were incubated with the labeled substrate for the indicated time points to allow incorporation under steady-state activation conditions. Cells were rapidly harvested, washed with NaCl 0.9%, and metabolism was quenched by the addition of cold extraction solvent. Cells were lysed with 1 ml of chloroform: MeOH: H2O (1:3:1) and centrifuged at 15,000 × g for 5 min at 4 °C. 450 µl of supernatant were collected and dried by using SpeedVac concentrator. Afterward, sample derivation was performed, briefly, 20 µl of 2% methoxyamine chloride was added and shaked for 2 h at 30 °C. Afterward, samples were derivatized with 20 µl BSTFA contain 1% TMCS and incubated by 1 h at 30 °C with shaked, after that the samples were analyzed by GC-MS. GC was performed using a DB5ms capillary column (J&W DB-5ms GC Column, 30 m, 0.25 mm, 0.25 µm, DuraGuard, 10 m; #122-5532G, Agilent Technologies). The GC inlet and GC-MS transfer line temperatures were maintained at 270 °C and 250 °C, respectively. The oven temperature gradient was programmed as follows: 70 °C (1 min); 70–295 °C at 12.5 °C/min; 295– 320 °C at 25 °C/min; 320 °C for 2 min. GC-MS data was imported in CDF-formatted files and analyzed by using DExSI (Data Extraction for Stable Isotope-labelled metabolites) (34), for mass isotopologues distributions (MIDs) and natural isotope abundance correction.

### 2.14 SCENITH, single-cell energetic metabolism by profiling translation inhibition protocol

To quantify cellular metabolic dependence of untreated and VitD-treated CD4^+^ T cells, SCENITH™ (Cat GO001050AF488 and GO006050AF647 acquired from www.gammaomics.com) was used to compare glucose and mitochondrial dependence and glycolytic capacity between cells. Briefly, activated 2x10^5^ CD4^+^ T cells with or without VitD were treated with DMSO, 2-Deoxy-Glucose (2-DG; 100 mM), Oligomycin (O; 1 µM), combination of 2-Deoxy-Glucose and Oligomycin (DGO) or BPTES (5µM) for 45 min at 37 °C and 5% of CO_2_. Then, Puromycin (10 µg/mL) was added during the last 15 min of the metabolic inhibitor treatment. After puromycin treatment, cells were washed with PBS and stained with a fluorescent cell viability dye (TONBO Biosciences Cat: 13-0865-T100) for 30 min at 4 °C in PBS. Then, cells were fixed and permeabilized using eBioscience FOXP3/transcription buffer (Thermo Fisher Scientific, Cat: 00-5523-00) following manufacturer instructions. Intracellular staining with anti-puromycin monoclonal antibody (clone R4743L-E8) with Alexa Fluor 488 were incubated for 60 min at 4 °C. Finally, data acquisition was performed using FortessaX20. Energetic metabolism parameters were calculated according to the formulas in Arguello R.J et al (35).

### 2.15 Determination of oxygen consumption rate under real-time conditions

The metabolic profile was assessed by real-time measurement of the oxygen consumption rate (OCR) using a Seahorse XFe96 extracellular flux analyzer (Agilent Technologies, Santa Clara, CA, USA) integrated with a Cytation 5/BioSpa 8 system (Agilent Technologies). Twenty-four h after cell sorting, cells were resuspended in unbuffered DMEM supplemented with 10 mM glucose, 4 mM glutamine, and 1 mM pyruvate (pH 7.4). Cells were then seeded into XFe96 V3 microplates pre-coated with poly-D-lysine to promote adhesion. To ensure a uniform monolayer, plates were centrifuged at 1,000 rpm for 5 min and subsequently incubated for 1 h at 37 °C in a CO_2_-free incubator to allow thermal equilibration and medium degassing. Prior to analysis, quality control was performed by brightfield microscopy using the Cytation 5 system. Mitochondrial function was evaluated using the Mito Stress Test, with sequential injections of the following electron transport chain (ETC) modulators: oligomycin (1 µM), to inhibit ATP synthase and determine ATP-linked respiration; FCCP (1 µM), an uncoupler used to achieve maximal respiratory capacity; and rotenone/antimycin A (1 µM), to inhibit complexes I and III, respectively, enabling quantification of non-mitochondrial respiration. Hoechst 33342 (1 µM) was co-injected with the final injection (Rot/AA) to facilitate downstream data normalization. Oxygen consumption rate (OCR) measurements were made using specific excitation and emission wavelengths for oxygen (532/650 nm) (36).

OCR values were normalized to cell number per well using Cell Integration software (Agilent Technologies). Normalization was based on *in situ* nuclear staining with Hoechst, with Hoechst fluorescence acquired using the Cytation 5 system and quantified by automated nuclear counting. Image-based quality control and OCR parameters were used to select wells exhibiting a uniform monolayer and appropriate responsiveness to ETC modulators. Wells displaying abnormal OCR profiles or non-uniform cell distribution were flagged and excluded from further analysis (37).

### 2.16 Statistical analysis

The data obtained from the flow cytometer were analyzed using FlowJo version 7.6, along with other data obtained from the microplate reader (SYNERGY 2, Biotek). Statistical analyses are mentioned in the figure legends and a P-value<0.05 was considered statistically significant. Data analysis was performed using GraphPad Prism Version 11.

## 3 Results

### 3.1 Vitamin D increase cell expansion and reduces glucose uptake in activated CD4^+^ T cells

**Figure 1:**
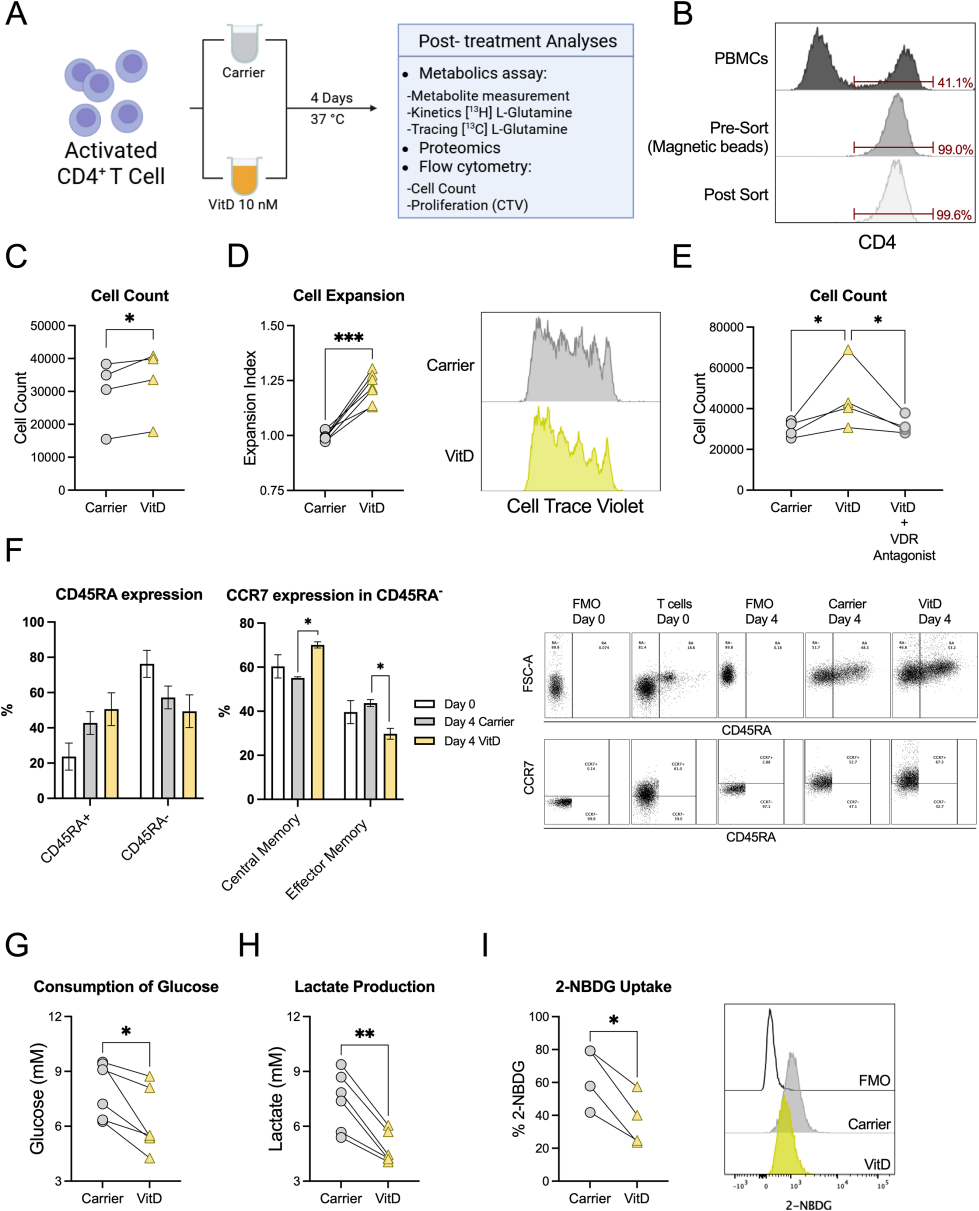
Vitamin D reduces glycolytic activity and increases initial cell expansion in activated CD4^+^ T cells. **(A)** Schematic representation of the experimental workflow. CD4^+^ T lymphocytes were isolated and cultured under carrier or VitD-treated conditions for 4 days, followed by flow cytometry, metabolite analysis, and proteomic profiling. **(B)** CD4^+^ T lymphocyte purity after isolation was confirmed by measuring CD4 expression in PBMCs, CD4^+^ T cells after magnetic column enrichment and after cell sorting. **(C)** Normalized cell counts determined by bead-based flow cytometry in CD4^+^ T treated and untreated with VitD (Paired t-test; N = 4). **(D)** Cell expansion values and representative histograms were obtained from Flowjo proliferation parameters in CD4^+^ T treated and untreated with VitD (Paired t-test; N = 7). **(E)** Normalized cell counts determined by bead-based flow cytometry in CD4^+^ T untreated and treated with VitD in the presence of VDR antagonist (1way ANOVA with Holm-Šídák’s multiple comparisons test; N = 4). **(F)** Representative dot plots and percentages of T cell subsets at the begging and at the end of the culture based on CD45RA and CCR7 expression (2way ANOVA with Šídák’s multiple comparisons test; N = 3). **(G)** Glucose consumption calculated as the difference between initial and final glucose concentrations in the culture medium after 4 days (Paired t test; N = 6). **(H)** Lactate production measured in culture supernatants after 4 days (Paired t test; N = 6). **(I)** Representative histogram showing gating strategy and fluorescence intensity of 2-NBDG uptake, followed by quantification of 2-NBDG positive cells (Paired t test; N = 4). Significance: p< 0.05 (*); p< 0.01 (**); p< 0.001 (***).

VitD has been widely associated with immunosuppressive functions and metabolic reprogramming in CD4^+^ T cells. We previously demonstrated that VitD increases VDR expression following T cell activation (Chaus et al., 2022, Extended Data Figure 1) (38), modulates T helper cell repertoires and enhances cell counts and cell division rates (Fraga et al., 2021, Figure 2) (7). In contrast, other studies have reported that VitD suppresses glycolysis in activated CD4^+^ T cells between 2 and 4 days after anti-CD3/CD28 stimulation (14). To further investigate this apparent discrepancy, we evaluated the immunometabolic effects of VitD in anti-CD3/CD28-activated CD4^+^ T lymphocytes cultured for 4 days in the presence or absence of physiological concentrations of VitD (1,25-dihydroxyvitamin D, 10 nM) (**Figure 1A**). CD4^+^ T cell enrichment and purity were confirmed by CD4 expression in all experiments (**Figure 1B**). VitD treatment significantly increased total CD4^+^ T cell numbers (**Figure 1C**) and cell expansion (**Figure 1D**). Importantly, pharmacological inhibition of VDR significantly reduced cell counts, confirming the requirement of VitD-VDR signaling for this effect (**Figure 1E**). Phenotypic analysis revealed that VitD increased the frequency of central memory T cells after 4 days in culture without significantly altering the distribution of CD45RA^+^ and CD45RA^−^ populations (**Figure 1F**). To evaluate metabolic activity, culture supernatants were analyzed for glucose consumption and lactate production, while glucose uptake was assessed using the fluorescent glucose analog 2-NBDG. VitD-treated cultures exhibited significantly reduced glucose consumption (**Figure 1G**), lower extracellular lactate accumulation (**Figure 1H**), and decreased glucose uptake (**Figure 1I**). Together, these findings demonstrate that the VitD-mediated increase in CD4^+^ T cell expansion occurs despite reduced glycolytic activity, suggesting that alternative metabolic pathways support cellular proliferation and energetic demands under these conditions.

### 3.2 Vitamin D enhances glutaminolytic and redox-associated pathways in activated CD4^+^ T cells

**Figure 2:**
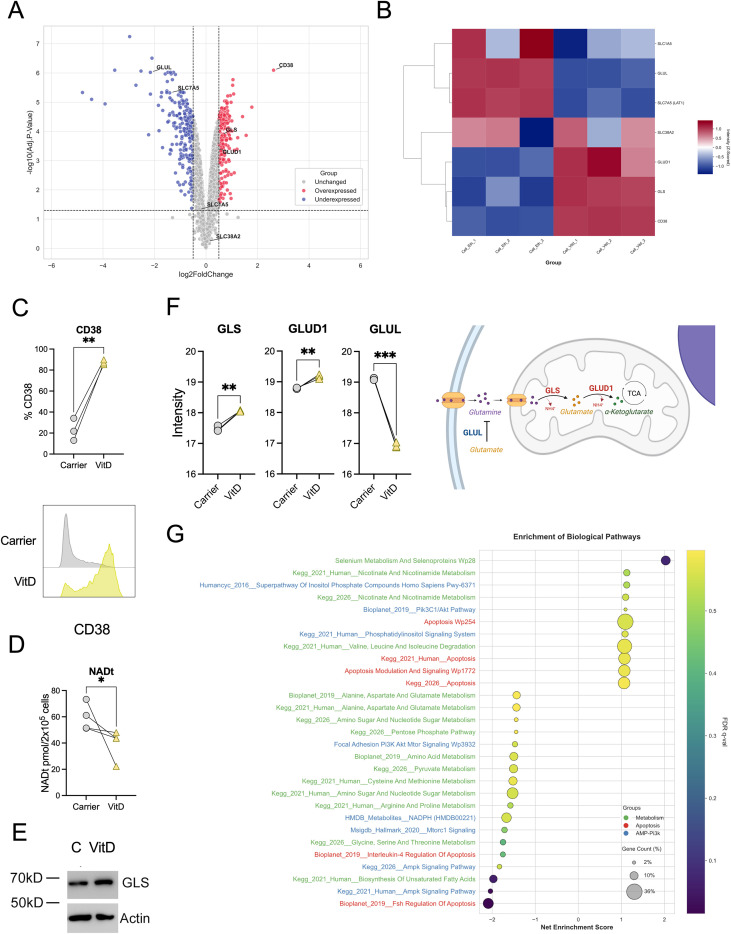
Vitamin D induces a glutaminolysis associated proteomic profile in activated CD4^+^ T cells. **(A)** Volcano plot of proteomic showing 2,756 quantified proteins, of which 449 were differentially expressed between VitD and carrier conditions (adjusted *p* ≤ 0.05; |log2FC| > 0.5), including 212 upregulated and 237 downregulated proteins (N = 3). **(B)** Clustermap of glutaminolysis related enzymes displaying relative expression (Z-Score normalization) between control and VitD-treated group (N = 3). **(C)** Validation of proteomics by measuring the expression of CD38 and **(D)** total NAD in CD4^+^ T cells in response to VitD (Paired t test; N = 3). **(E)** Western blot of GLS in control and VitD-treated T cells. **(F)** Schematic representation and intensity of glutaminolysis-associated metabolic pathways indicating metabolites, enzymes and cellular localization (Paired t test; N = 3). **(G)** Pre-ranked Gene Set Enrichment Analysis (GSEA) performed with the *prerank* module of the GSEApy library. Significance: p< 0.05 (*); p< 0.01 (**); p< 0.001 (***).

To investigate alternative metabolic pathways supporting CD4^+^ T cell expansion in the presence of VitD, we performed proteomic profiling of activated CD4^+^ T cells cultured with or without VitD. A total of 2,756 proteins were identified (**Supplementary Table 1**), of which 449 were differentially expressed between VitD and carrier (ethanol) conditions (adjusted *p* ≤ 0.05; |log2FC| > 0.5), including 212 upregulated and 237 downregulated proteins (**Figure 4A**; **Supplementary Table 2**). Comparative proteomic analysis revealed significant modulation of proteins associated with glutamine metabolism and increased expression of CD38 (**Figure 2B**; **Supplementary Table 3**). Specifically, VitD increased the expression of glutaminase (GLS) and glutamate dehydrogenase 1 (GLUD1), while glutamine synthetase (GLUL) expression was reduced (**Figure 2B**, **Supplementary Table 4**), suggesting enhanced glutamine catabolism toward glutaminolysis. In contrast, proteins associated with glutamine transport showed limited changes. Expression of SLC1A5 (ASCT2) and SLC38A2 (SNAT2) remained unchanged, whereas SLC7A5 (LAT1) expression was significantly reduced in the presence of VitD (**Figure 2B**; **Supplementary Table 4**). Increased CD38 expression was further confirmed by flow cytometry (**Figure 2C**) and functional analysis of intracellular NAD levels (**Figure 2D**), whereas GLS increment was confirmed by western blot (**Figure 2E**). A schematic representation of glutaminolysis together with GLS, GLUD1 and GLUL protein expression intensity is shown in **Figure 2F**. Pathway enrichment analysis identified three major categories differentially regulated by VitD treatment: metabolism (in green), apoptosis (in red), and AMPK-PI3K (in blue) signaling pathways. Within metabolic pathways, Selenium Metabolism and Selenoproteins and Nicotinate and Nicotinamide Metabolism were positively enriched in VitD-treated cells, whereas biosynthesis of unsaturated fatty acids was negatively enriched (**Figure 2G**; **Supplementary Table 5**). Additional metabolic pathways associated with amino acid metabolism, pyruvate metabolism, and the pentose phosphate pathway were also negatively enriched by VitD (**Figure 2G**; **Supplementary Table 5**). Regarding cell survival pathways, apoptosis-related pathways were enriched in VitD-treated cells, whereas apoptosis regulation mediated by FSH and IL-4 signaling was negatively enriched (**Figure 2G**). In parallel, AMPK signaling was reduced, while pathways associated with inositol phosphate metabolism, PI3K/Akt signaling, and phosphatidylinositol signaling were positively enriched in the presence of VitD (**Figure 2G**). Collectively, these findings indicate that VitD induces a broad metabolic rewiring characterized by suppression of canonical glycolytic and biosynthetic pathways together with enrichment of glutaminolysis-associated enzymes, redox-related pathways, and phosphoinositide signaling. Although VitD increased CD4^+^ T cell expansion, the precise contribution of glutaminolysis to this phenotype remained unclear, particularly because major glutamine transporters were not upregulated at the proteomic level. Therefore, we next evaluated whether VitD directly modulates glutamine uptake and transport efficiency in activated CD4^+^ T cells.

### 3.3 Vitamin D enhances glutamine uptake and transport efficiency in activated CD4^+^ T cells

**Figure 3:**
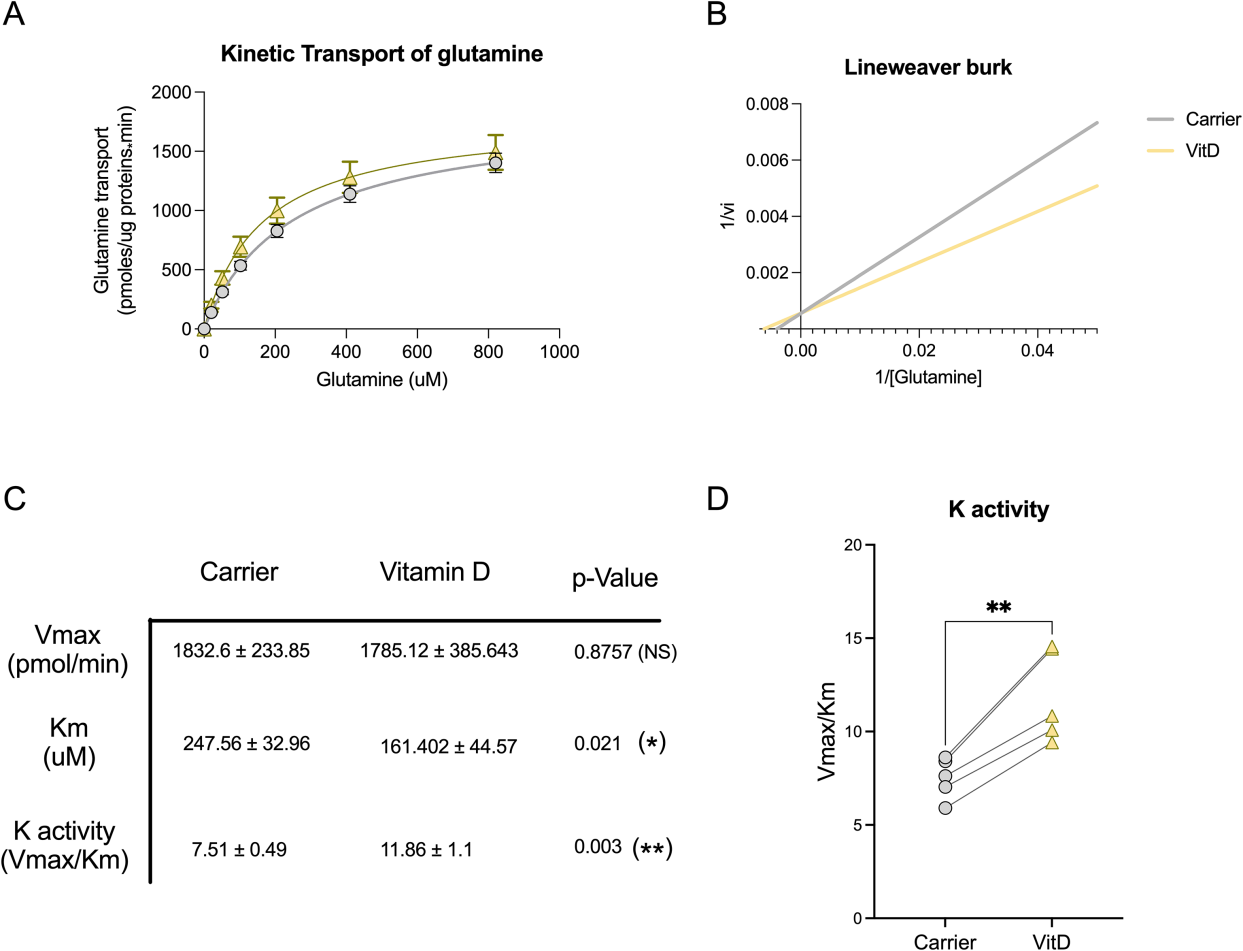
Vitamin D increases the affinity and functional efficiency of glutamine transport in activated CD4^+^ T lymphocytes. **(A)** Michaelis-Menten equation curve generated by direct fitting of glutamine uptake kinetic under carrier and VitD-treated conditions. Individual donor-derived kinetic curves and donor-specific Vmax and Km values are shown in **Supplementary Figure 1**. **(B)** Lineweaver-Burk transformation of the Michelis-Menten equation used to obtain linearized estimates of Vmax and Km. **(C)** Table summarizing Vmax, Km, and the Vmax/Km ratio for control and VitD treated conditions (Paired t test; N = 3). **(D)** Graphical representation of the Vmax/Km ratio plotted for both conditions (Paired t test; N = 5). Significance: p< 0.05 (*); p< 0.01 (**).

To assess whether VitD alters the kinetic properties of glutamine transport, uptake assays were performed using tritiated glutamine. After 4 days of culture, CD4^+^ lymphocytes were incubated with increasing concentrations of unlabeled glutamine (0-250 µM) and a fixed concentration of tritiated glutamine (5µCi 3H glutamine/mL) at 37 °C for one min. The uptake data were fitted to the Michaelis-Menten equation (**Figure 3A**, **Supplementary Figure 1**). The resulting kinetic parameters were as follows: control cells, Vmax = 1832.6 ± 233.85 pmol/min and Km = 247.56 ± 32. µM: VitD treated cells, Vmax = 1785 ± 385 pmol/min and Km = 161.4 ± 44.6 µM. The significant decrease in Km in the presence of VitD indicates an increased affinity of the glutamine transporter system. These findings were consistent with the Lineweaver-Burk analysis (**Figures 3B, C**). To integrate both parameters and evaluate overall transport performance, the Vmax/Km ratio (an indicator of transport efficiency) was calculated. The ratio increased significantly in VitD-treated cells compared with control cells (**Figures 3D**), indicating a higher functional transport efficiency, particularly at physiologically relevant glutamine concentrations. In summary, these data indicate that VitD enhances the affinity and functional efficiency of glutamine transport in activated CD4^+^ T lymphocytes. Next, the conversion of glutamine to glutamate and alpha-ketoglutarate (α-Kg) was measured to confirm VitD-mediated glutaminolysis.

### 3.4 Vitamin D enhances glutaminolytic flux according to isotopic tracing analysis

**Figure 4:**
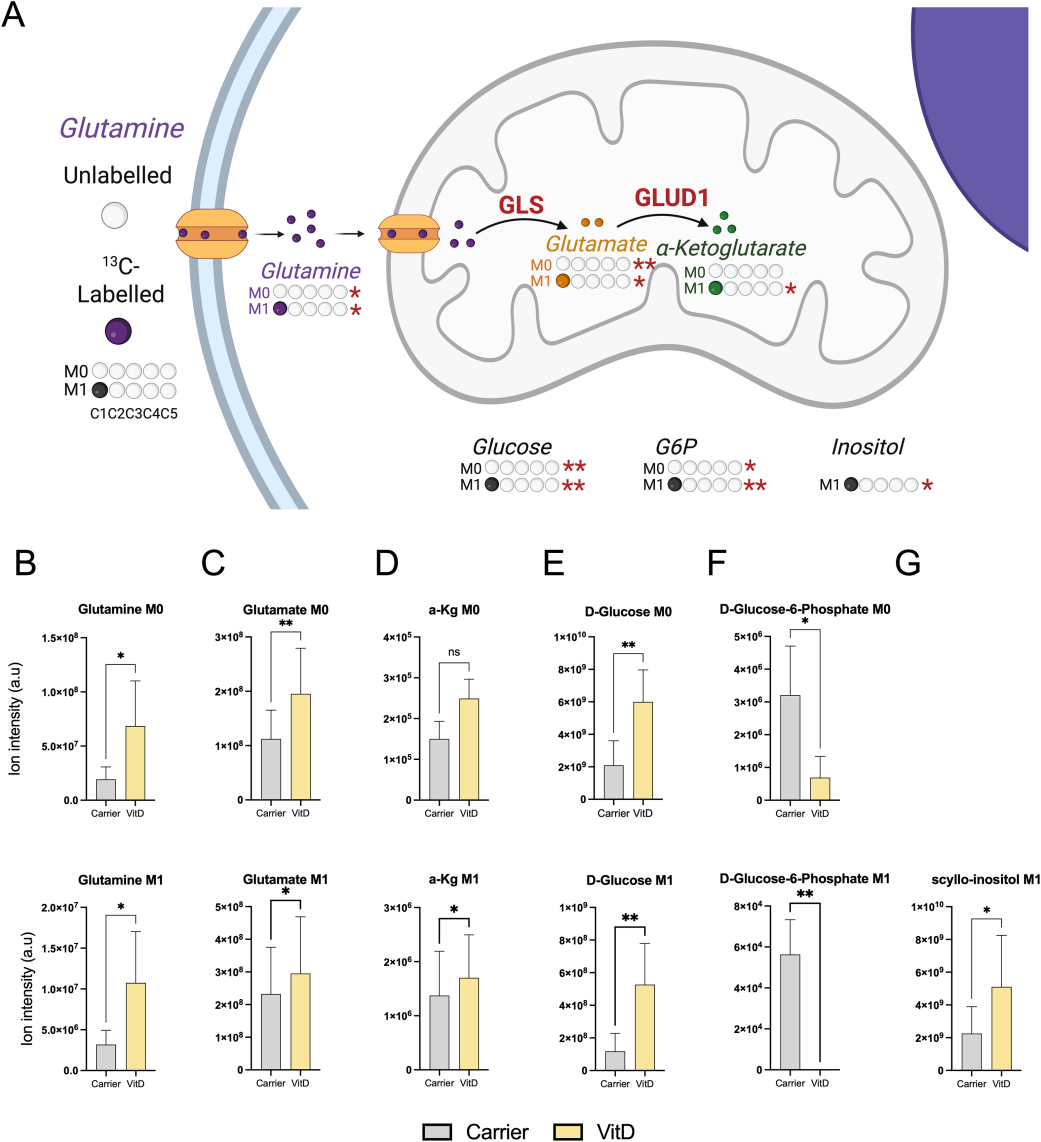
Isotopic tracing reveals enhanced glutaminolysis flux and reduced glycolytic commitment in Vitamin D treated CD4^+^ T cells. **(A)** Schematic representation of [1-^13^C]-L-glutamine uptake and intracellular metabolic conversion leading to tricarboxylic acid (TCA) cycle intermediates. Ion intensity of **(B)** glutamine isotopologues, **(C)** glutamate isotopologues, **(D)** α-ketoglutarate isotopologues, **(E)** D-glucose isotopologues and **(F)** glucose-6-phosphate isotopologues at M0 and M1 measured under carrier and VitD-treated conditions (Paired t test; N = 6). **(G)** Ion intensity of scyllo-inositol isotopologue at M1 measured under carrier and VitD-treated conditions (Paired t test; N = 6). Significance: p< 0.05 (*); p< 0.01 (**).

To determine the metabolic fate of glutamine following its uptake by CD4^+^ lymphocytes after 4 days of activation, we performed isotopic tracing using [1-^13^C]-L-glutamine during the final 24 h of incubation. This tracing allowed us to delineate the trajectory of glutamine uptake, its conversion to glutamate, and subsequent transformation into α-Kg, as illustrated in the schematic **Figure 4A**. In cells treated with VitD, there was a significant increase in both unlabeled (M0) and labeled (M1) isotopologues of glutamine (**Figure 4B**), glutamate (**Figure 4C**), and α-Kg (**Figure 4D**), indicating that glutaminolysis is active. Concomitantly, VitD treatment also increased in both the total (M0) and labeled (M1) isotopologues of D-glucose (**Figure 4E**), suggesting potential gluconeogenesis derived from glutamine carbons. In addition, levels of glucose-phosphate (G6P) were significantly reduced across all isotopologues (M0-M1, **Figure 4F**), reflecting a decline in glycolytic flux. Despite the reduced G6P pool and the absence of detectable ¹³C enrichment in this metabolite, both glucose (**Figure 4E**) and inositol (**Figure 4G**, **Supplementary Figure 2A**) displayed ¹³C labeling following [1-¹³C]-glutamine tracing. Importantly, the lack of early increases in pyruvate, malate, or citrate excludes a major contribution of reverse TCA flux or gluconeogenic routing from glutamine-derived carbon (**Supplementary Figure 2B**). Instead, this labeling pattern is consistent with a high-flux, low-pool model in which G6P functions as a transient metabolic node that is rapidly consumed rather than accumulated. Under these conditions, glutamine metabolism does not provide carbon directly to inositol but rather sustains the energetic and redox environment required for selective routing of glucose-derived carbon toward inositol biosynthesis. Consequently, inositol acts as a downstream metabolic sink that integrates low-level carbon flux over time, explaining its accumulation and isotopic enrichment despite reduced G6P abundance.

### 3.5 Glutamine uptake and conversion to glutamate mediate the increment of cell counts and proliferation indexes of Vitamin D-activated CD4^+^ T cells

**Figure 5:**
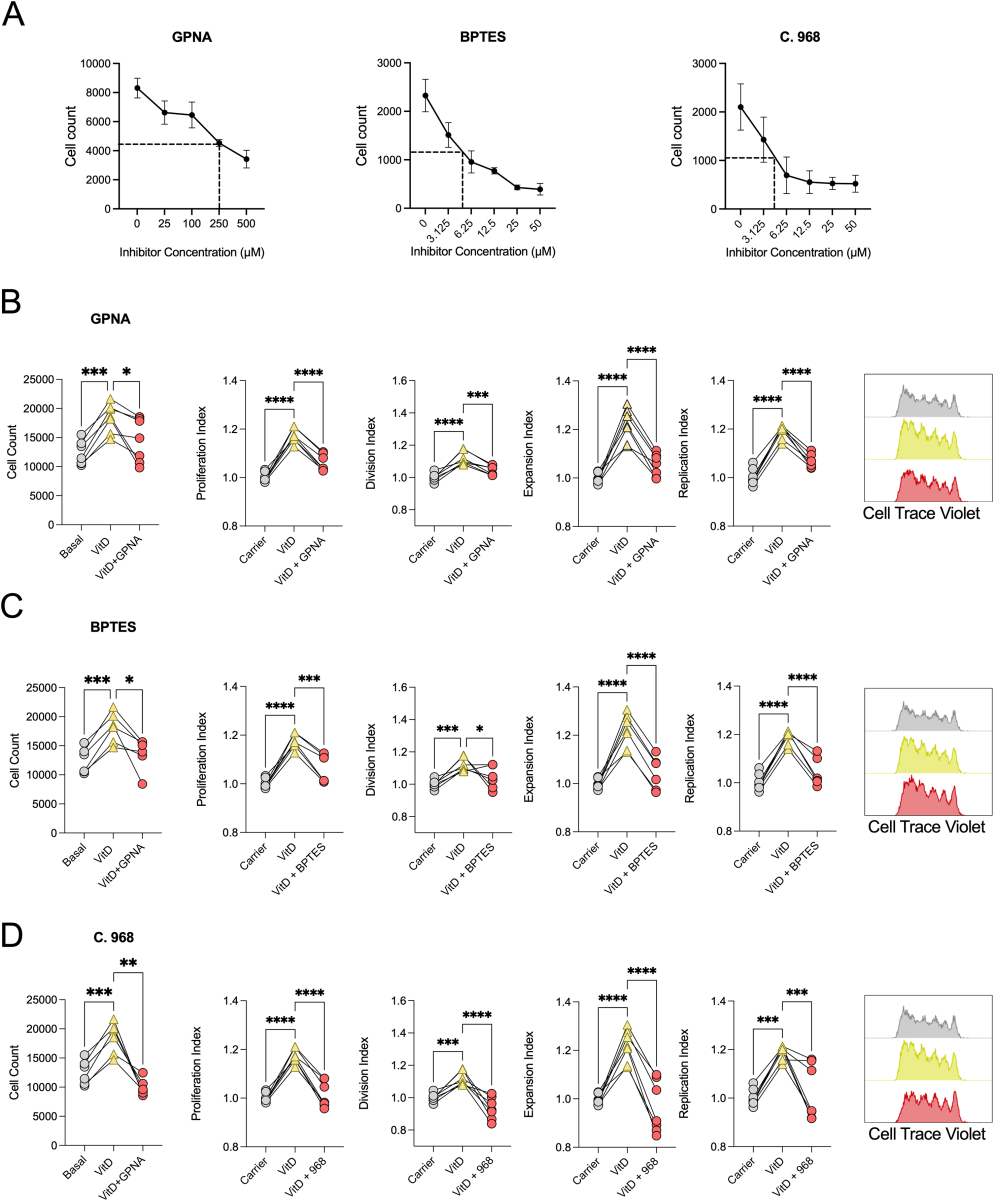
Glutamine uptake and glutamate production are required for Vitamin D driven proliferation of activated CD4^+^ T cells. **(A)** Dose-response curves showing normalized cells count as a function of increasing concentrations (µM) of the glutamine transport inhibitor GPNA and the glutaminase inhibitors BPTES and compound 968. (Mean with Standard Deviation; N = 4). Cell count, proliferation index, division index, expansion index, replication index and representative histograms comparing basal activated CD4^+^ T cells, VitD-treated CD4^+^ T cells, and VitD-treated CD4^+^ T cells plus **(B)** GPNA or **(C)** BPTES or **(D)** C.968 (RM one-way ANOVA with Tukey´s multiple comparisons test; N = 6). Proliferation indices: Proliferation index, defined as the total number of division divided by the number of cells that entered division; Division index, defined as the total number of divisions divided by the number of cells at the start of culture; Expansion index, defined as the total number of cells divided by the number of cells at the start of culture; and Replication index, defined as the total number of divided cells divided by the number of cells that entered division. Significance p< 0.05 (*); p< 0.01 (**); p< 0.001 (***); p< 0.0001 (****).

After observing that the glutaminolytic pathway is favored in activated CD4^+^ T lymphocytes cultured in the presence of VitD, we next assessed the effect of its inhibition through cell counting. Dose-response curves were performed to determine the optimal concentration of the inhibitors to be used (**Figure 5A**). The inhibitors were added after 2 days of activation. This time point was selected based on our previous data following the kinetics of VDR (4), as the target receptor is known to be upregulated following cell activation (7, 38 [Extended Data **Figure 1**]). Thus, the treatment was applied when receptor availability was highest, allowing for a more specific and physiologically relevant assessment of the inhibitor’s effects. The concentration of glutamine inhibitors that reduced cell counts by approximately 50% were selected. The optimal concentration for the glutamine transport inhibitor GPNA was 250 µM, while for the glutaminase inhibitors (BPTES and compound 968), the optimal concentration was 5 µM. To better understand how glutamine transporter impacts cellular proliferation, we performed both cell counting and proliferation assay by flow cytometry. These analyses revealed that inhibition of glutamine uptake with GPNA (250 µM) led to a significant reduction in cell count as well as in proliferation, division, expansion and replication indices when compared with cell treated with VitD alone (**Figure 5B**). Together, these findings suggest that glutamine uptake plays a critical role in the proliferation of these cells, and that blocking its entry impairs their ability to grow properly. To evaluate the impact of glutamate conversion on the proliferation of activated CD4^+^ T lymphocytes in the presence of VitD, the glutaminase inhibitors BPTES (**Figure 5C**) and compound 968 (**Figure 5D**) were added after 2 days of culture. Both compounds block the enzyme that catalyzes the conversion of glutamine to glutamate, a key step in glutaminolysis. Treatment with VitD with either BPTES (**Figure 5C**) or compound 968 (**Figure 5D**) significantly reduced cell count, and proliferation indices compared to VitD alone. These results indicate that both inhibitors impair cellular proliferation and highlight that not only glutamine uptake, but also its conversion to glutamate, is critically important for cell proliferation.

### 3.6 Vitamin D inhibited oxidative phosphorylation in T CD4^+^ lymphocytes, without inducing mitochondrial or cell stress

**Figure 6:**
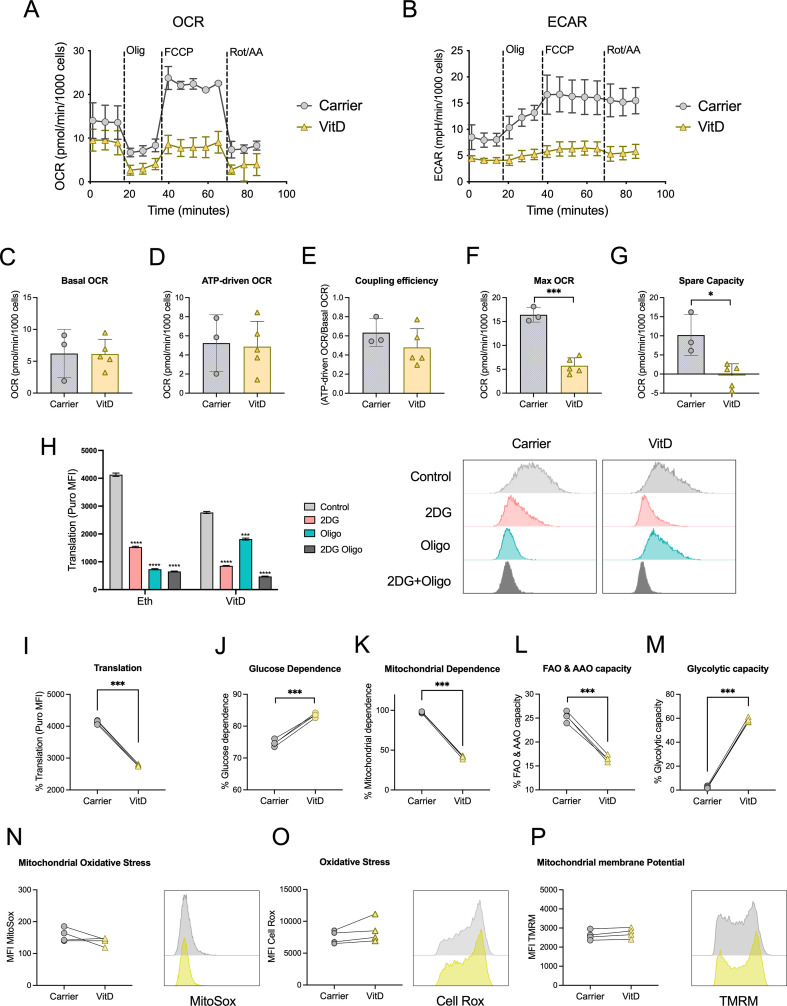
Vitamin D inhibited oxidative phosphorylation in T CD4^+^ lymphocytes, without inducing mitochondrial or cell stress. **(A)** Oxygen consumption rate (OCR) and **(B)** Extracellular acidification rate (ECAR) of CD4^+^ T treated and untreated with VitD. **(C)** Basal OCR, **(D)** ATP-linked OCR **(E)** coupling efficiency, **(F)** maximal mitochondrial respiration and **(G)** Spare respiratory capacity. (For B-G, Bar charts with mean and standard deviation and individual values; Unpaired t test; N = 3 carrier and N = 5 VitD). **(H)** Representative histogram and bar chart of protein translation levels by puromycin MFI in CD4^+^ T treated and untreated with VitD, (Bar charts with mean and standard deviation, 2-way ANOVA with Tukey´s multiple comparisons test; N = 4). **(I)** Basal protein translation levels by puromycin MFI between CD4^+^ T treated and untreated with VitD, (Paired t test; N = 4). Comparison of **(J)** Glucose dependence, **(K)** Mitochondrial dependency **(L)** FAO and AAO capacity, and **(M)** Glycolytic capacity between CD4^+^ T treated and untreated with VitD (For J-M, Paired t test; N = 4). Comparison of **(N)** mitochondrial oxidative stress, **(O)** cellular oxidative stress and **(P)** mitochondrial membrane potential between CD4^+^ T treated and untreated with VitD (For N-P, Paired t test; N = 4). Significance: p< 0.05 (*); p< 0.001 (***).

To evaluate whether VitD improves the respiration profile via glutaminolysis, oxygen consumption rate (OCR), and translation of proteins in the presence or absence of 2-Deoxy-Glucose and oligomycin by SCENITH was measured. **Figure 6A** shows the respiration profile of CD4^+^ T cells treated with carrier or VitD, and **Figure 6B** shows media acidification using the same conditions (**Figures 6A, B**, **Supplementary Figure 3**). The data indicate that VitD does not alter basal oxygen consumption rate (OCR) (**Figure 6C**), ATP-linked OCR (**Figure 6D**), or coupling efficiency (**Figure 6E**). In contrast, VitD significantly reduces maximal mitochondrial respiration (**Figure 6F**) and spare respiratory capacity (**Figure 6G**). These results suggest that VitD-treated CD4^+^ T cells exhibit a diminished maximal electron transport capacity derived from substrate oxidation, thereby limiting their ability to meet increased energetic demands. Metabolic profiling using SCENITH (Argüello et al., 2020) (35) (**Figure 6H**), indicated that VitD inhibits protein synthesis in CD4^+^ T cells (**Figure 6I**). Interestingly, protein synthesis relies predominantly on glycolysis, and to a much lesser extend on oxidative phosphorylation in the presence of VitD (**Figures 6J–M**), indicating that glutaminolysis is not fueling the TCA cycle, opting for a glycolytic pathway. Of note, inhibition of glutaminolysis did not affect protein synthesis in the absence of VitD (**Supplementary Figure 4**). However, treatment with VitD in combination with BPTES increased protein synthesis compared with VitD alone, suggesting that VitD-driven glutamine metabolism may preferentially support anabolic rather than catabolic processes in CD4^+^ T cells at day 4. This idea is supported by the observation of [1-^13^C]-L-glutamine in glucose and inositol. Finally, malfunction of mitochondria by oxidative stress in the presence of VitD was discarded, since mitochondrial oxidative stress (**Figure 6N**), cellular oxidative stress (**Figure 6O**) and mitochondrial membrane potential (**Figure 6P**) were no affected by the presence of VitD.

### 3.7 Vitamin D post 7 days of culture reduced the proliferation of T CD4^+^ Lymphocytes, which can be rescued by addition of glutamine

**Figure 7:**
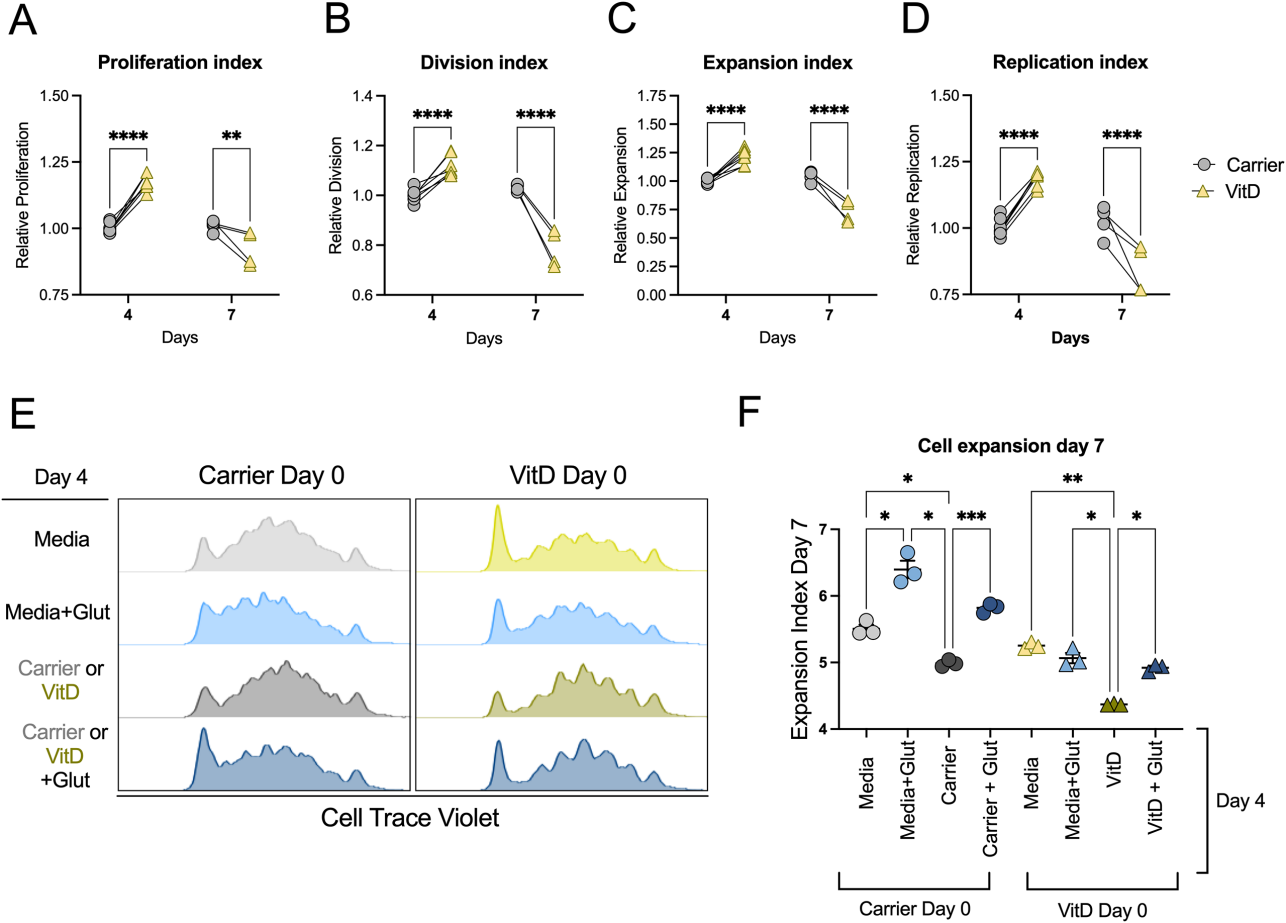
Vitamin D exerts a time-dependent biphasic effect of CD4^+^ T cell proliferation. **(A)** Relative Proliferation index, **(B)** Division index, **(C)** Expansion index and **(D)** Replication index at day 4 and 7 of culture comparing carrier and VitD-treated conditions, normalized to the mean of the corresponding control group (For **(A-D)**, 2way ANOVA with mixed effect model and Šídák’s multiple comparisons test; N = 8 day 4, N = 4 day 7). **(E)** Representative histograms and **(F)** Expansion index at day 7 of CD4^+^ T treated and untreated with VitD from day 0, with and without addition of VitD and/or glutamine at day 4 (RM one-way ANOVA with Tukey’s multiple comparisons test; N = 3). Significance: p< 0.05 (*); p< 0.01 (**); p< 0.001 (***); p< 0.0001 (****).

Our data have highlighted the inhibition of classical metabolic pathways and protein synthesis in CD4^+^ T cells with VitD, despite observing an increment in glutaminolysis, proliferation and cell count at day 4 post activation. To determine whether these pathways affect CD4^+^ T cells long term, we follow the culture until day 7. The temporal analysis of T cells proliferation revealed a biphasic dynamic in response to VitD treatment. During the first four days of culture, cells exposed to VitD showed a significant increase in division, proliferation, expansion, and replication indices compared with control cells. To ensure accurate comparison across time points, proliferation indices were normalized to the corresponding day of culture, providing a clearer representation of the temporal dynamics observed. By day 7, proliferation index (**Figure 7A**), division index (**Figure 7B**), expansion index (**Figure 7C**) and replication index (**Figure 7D**) were completely reversed, with a marked reduction in all proliferative parameters relative to the untreated group. This was observed independent of glutamine and VitD addition at day 4 (**Figures 7E, F**). However, when VitD and glutamine were added in combination at day 4, cell counts increased in comparison with the addition of VitD alone, suggesting that replacement of VitD in the presence of glutamine re-activated the proliferative response observed in the first 4 days (**Figure 7F**). These findings indicate that the effect of VitD on T lymphocyte proliferation is time-dependent, characterized by an initial stimulatory phase followed by a subsequent suppressive phase if both VitD and glutamine are not maintained.

## 4 Discussion

VitD has traditionally been viewed as an immunoregulatory hormone controlling T cell activation, differentiation, and proliferation. However, increasing evidence indicates that its function extends beyond classical immune regulation, positioning VitD as an active modulator of cellular metabolism during immune activation (2). In line with this concept, our findings support a role for VitD as an immunometabolic regulator capable of reprogramming nutrient utilization and central metabolic pathways in activated CD4^+^ T cells. Activated CD4^+^ T cells are classically characterized by a strong reliance on aerobic glycolysis to sustain rapid clonal expansion (39). Nonetheless, our data demonstrate that early proliferation is enhanced in the presence of VitD despite a marked reduction in glycolytic activity. This observation indicates the engagement of alternative metabolic programs capable of supporting early T cell activation and challenges the notion that glycolysis is the sole metabolic driver of early CD4^+^ T cell expansion. The increased clonal expansion observed under VitD exposure, together with reduced glycolytic flux, suggests a shift in the hierarchy of metabolic substrate utilization. Our results indicate that glutaminolysis emerges as a central pathway supporting both the energetic and biosynthetic demands of early activation, allowing partial uncoupling of proliferation from exclusive dependence on aerobic glycolysis. While previous studies have reported VitD-induced suppression of glycolysis without clear activation of compensatory pathways, our findings suggest that glutaminolysis may become metabolically relevant under specific activation conditions and temporal windows (14). Consistent with this model, we observed increased glutamine uptake in VitD-treated CD4^+^ T cells.

Previous studies have shown that VitD can modulate amino acid transporter systems in non-immune tissues, such as the placenta, enhancing amino acid availability, suggesting that VitD can influence nutrient transport across diverse cellular contexts (40). In the present study, kinetic analyses indicated a reduction in the Km of glutamine transport without changes in maximal transport velocity, suggesting enhanced transporter affinity rather than increased transport capacity. This may explain the absence of detectable changes in glutamine transporter expression in our proteomic analysis and supports the notion that VitD regulates glutamine transport predominantly at a functional level rather than through transcriptional control. Although VitD signaling is classically associated with VDR-mediated transcriptional regulation, it can also activate signaling pathways involved in post-translational modifications, including STAT3 and c-Jun (38, 41). Notably, previously described post-translational regulation of glutamine transporters has primarily been linked to changes in transport capacity (42, 43). The preferential modulation of transporter affinity observed here suggests the existence of an alternative regulatory mechanism, potentially induced by VitD, that fine-tunes glutamine transport kinetics in activated CD4^+^ T cells.

A shift toward increased glutaminolytic dependence is expected to alter intracellular glutamine availability due to increased utilization. In this context, glutamine is required not only for anaplerotic fueling of the tricarboxylic acid cycle but also for nucleotide biosynthesis, redox balance, and protein synthesis, substantially increasing metabolic demand during activation (17). A reduction in free intracellular glutamine would favor an increased concentration gradient, promoting net glutamine influx independently of transporter expression changes. Such demand-driven regulation is consistent with the increased apparent affinity detected in our transport analyses. Supporting this interpretation, ¹³C-glutamine tracing revealed increased labeling of glutamine-derived metabolites, including glutamate and α-ketoglutarate, indicating enhanced glutaminolytic flux and increased anaplerotic input. Importantly, this metabolic shift does not appear to be secondary to glucose limitation but rather reflects VitD-driven metabolic reprogramming favoring glutamine as a predominant carbon source during early CD4^+^ T cell activation.

Analysis of proliferation kinetics revealed a temporal shift in the response to VitD, with reduced proliferation emerging after prolonged culture. This suggests that VitD-mediated metabolic activation is dynamically regulated over time. Early activation may involve mTOR-dependent metabolic support, which has been shown to play a central role in sustaining anabolic metabolism during T cell activation (44), whereas sustained proliferation likely requires regulatory mechanisms to prevent excessive immune activation. One potential mechanism involves induction of CYP24A1, the enzyme responsible for VitD catabolism (45), which limits the magnitude and duration of VitD signaling. Importantly, CYP24A1 expression integrates VitD receptor-dependent transcription with non-genomic MAPK-mediated signaling, positioning it as a convergence point for regulatory and metabolic cues that may promote the establishment of a more regulatory cellular environment over time (46). Intriguingly, large-scale proteomic interaction datasets curated in public databases have identified a potential association between the glutamine transporter ASCT2 and CYP24A1 (BioGRID interaction ID:112401), raising the possibility that VitD-dependent control of proliferation may be functionally linked to glutamine metabolism and suggesting the existence of a metabolic autoregulatory loop connecting glutaminolysis with VitD signaling and catabolism (47).

Our data suggest that VitD promotes a non-canonical immunometabolic program in activated CD4^+^ T cells in which cellular expansion is sustained despite reduced enrichment of glycolysis-associated pathways, pyruvate metabolism, amino acid metabolism and the pentose phosphate pathway. Instead, VitD appears to favor a glutaminolysis-dependent adaptive state characterized by enhanced metabolic homeostasis rather than classical catabolic energy production. Glutaminolysis is known to support proliferating T cells by providing carbon and nitrogen intermediates required for nucleotide, amino acid, and protein synthesis, independently of direct ATP generation through glycolysis or OXPHOS (48). In agreement with this, inhibition of glutaminolysis reduced VitD-associated cell expansion, supporting a functional role for glutamine metabolism in this phenotype. However, the absence of a concomitant increase in canonical glycolytic and biosynthetic pathways suggests that glutaminolysis under VitD conditions may preferentially sustain anabolic maintenance and cellular ess rather than a highly energetic effector program. This regulation is consistent with recent evidence of the VitD effects on breast cancer cells (49) and obese patients (50). Notably, VitD treatment enriched selenium metabolism and selenoproteins and nicotinate and nicotinamide metabolism, both strongly linked to antioxidant defense, NAD+ homeostasis, mitochondrial adaptation, and T-cell metabolic regulation. Together, these findings support the idea that VitD induces a survival-oriented metabolic rewiring characterized by energy conservation, redox control, stress resistance, and inflammatory restraint while still permitting cellular proliferation.

Together, these findings identify glutaminolysis as a key metabolic pathway supporting early CD4^+^ T cell activation under VitD exposure and highlight VitD as a dynamic regulator of T cell immunometabolism.

## Data Availability

The datasets presented in this study can be found in online repositories. The names of the repository/repositories and accession number(s) can be found in the article/**Supplementary Material**.
