# Supplementary material for: Vitamin D supports activated CD4+ T-cell proliferation through enhanced glutaminolysis
Source: Front Immunol. 2026 Jun 11;17:1791098. doi: 10.3389/fimmu.2026.1791098 (PMC13293794; doi:10.3389/fimmu.2026.1791098)
Supplement: Supplementary file 6 [file DataSheet1.pdf]

Supplementary Material

Vitamin D supports activated $CD4^+$ T-cell proliferation through enhanced glutaminolysis

Agustín A. Vera[1], Mauricio Hernandez[1,2], Ricardo A. Cartes[1,3], Faryd Llerena[1], Solange E. Cisterna[1], Romina A. Quiroga[1], Sergio A. Sanhueza[1], Camila P. Muñoz-Grez[1,4], Francisco Vergara[1], Daniel Moena[5] Pablo A. Alarcón[6], Rafael Burgos[6], Coralia I. Rivas[7], Elena Uribe[8], Rafael J. Argüello[9], Cristopher Almarza[10], Felix A. Urra[10], Francisco Tapia[11], Luciano Ferrada[12], Liliana I. Lamperti[1*] and Estefanía Nova-Lamperti[1*]

*** Correspondence:**

Estefanía Nova-Lamperti

enovalamperti@gmail.com, enova@udec.cl

Liliana Lamperti

llampert@udec.cl

1.1 Supplementary Figures

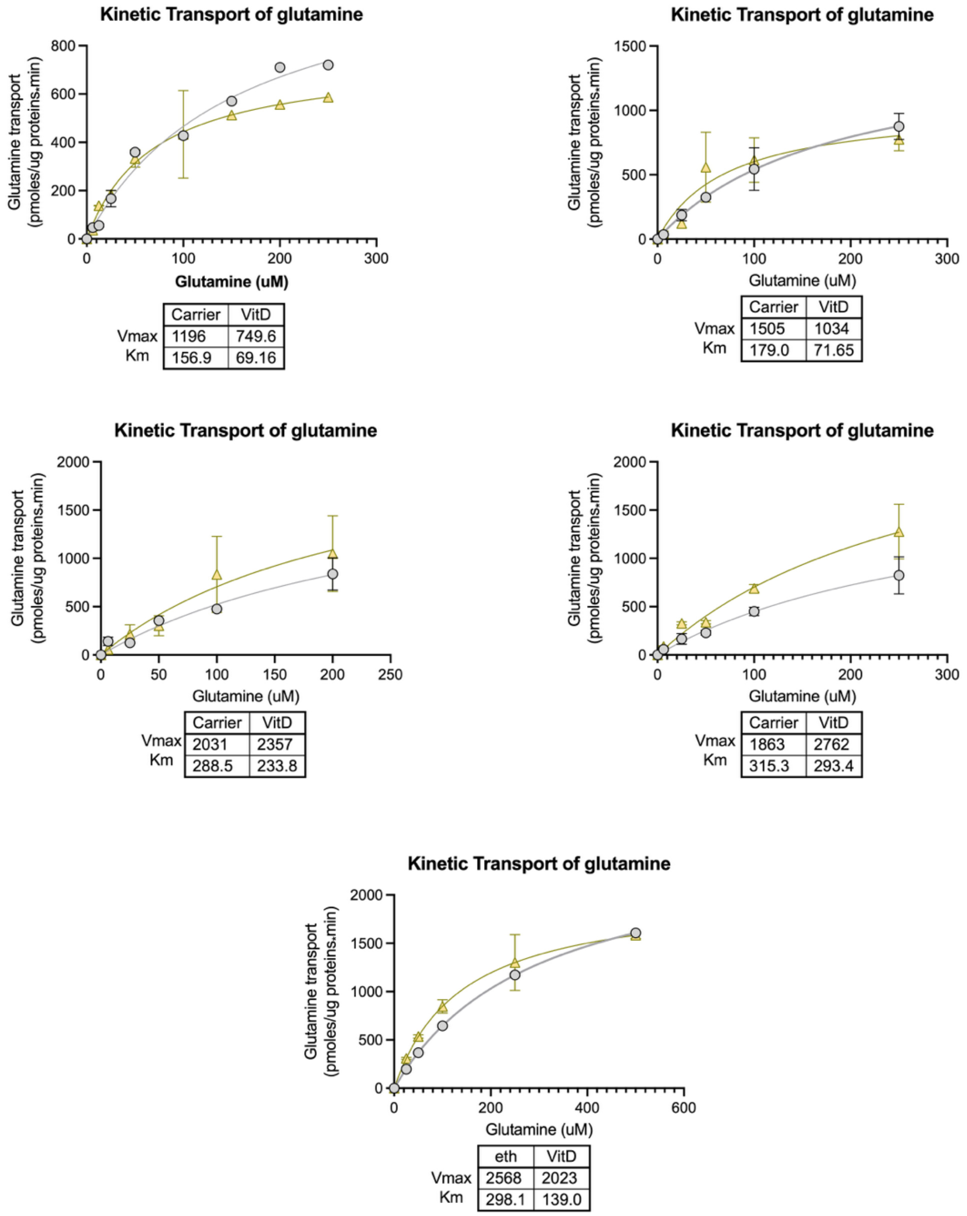

Supplementary Figure 1: Individual donor-derived kinetic analysis of glutamine transport in activated CD4⁺ T cells.

Michaelis-Menten uptake curves of glutamine transport obtained from individual donors. Activated $CD4^+$ T lymphocytes were cultured under control or VitD-treated conditions for 4 days and subjected to glutamine uptake assay using increasing concentrations of unlabeled glutamine and fixed concentration of tritiated glutamine. For each donor, uptake rates were fitted to the Michaelis-Menten equation, and the corresponding Vmax and Km values are indicated. No statistical comparisons were performed for individual donor curves. This figure illustrates inter-donor variability and supports the kinetic parameter summarized in Figure 2.

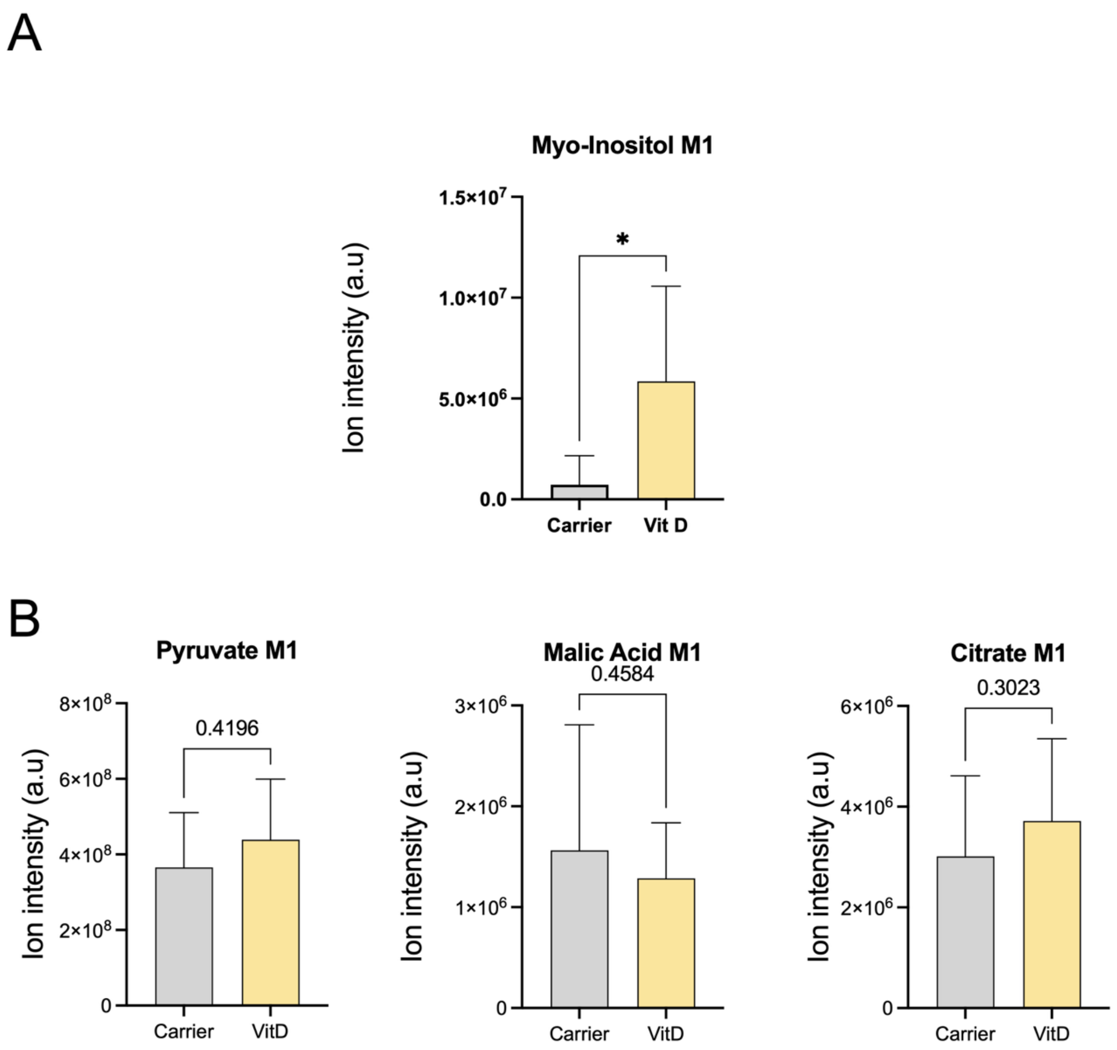

Supplementary Figure 2: Isotopic tracing of central carbon metabolism following [1-13C]-glutamine labeling.

(A) Significant 13C enrichment in myo-inositol after [1-13C]-glutamine tracing. (B) (A) 13C enrichment in pyruvate, malic acid and citrate after [1-13C]-glutamine tracing.

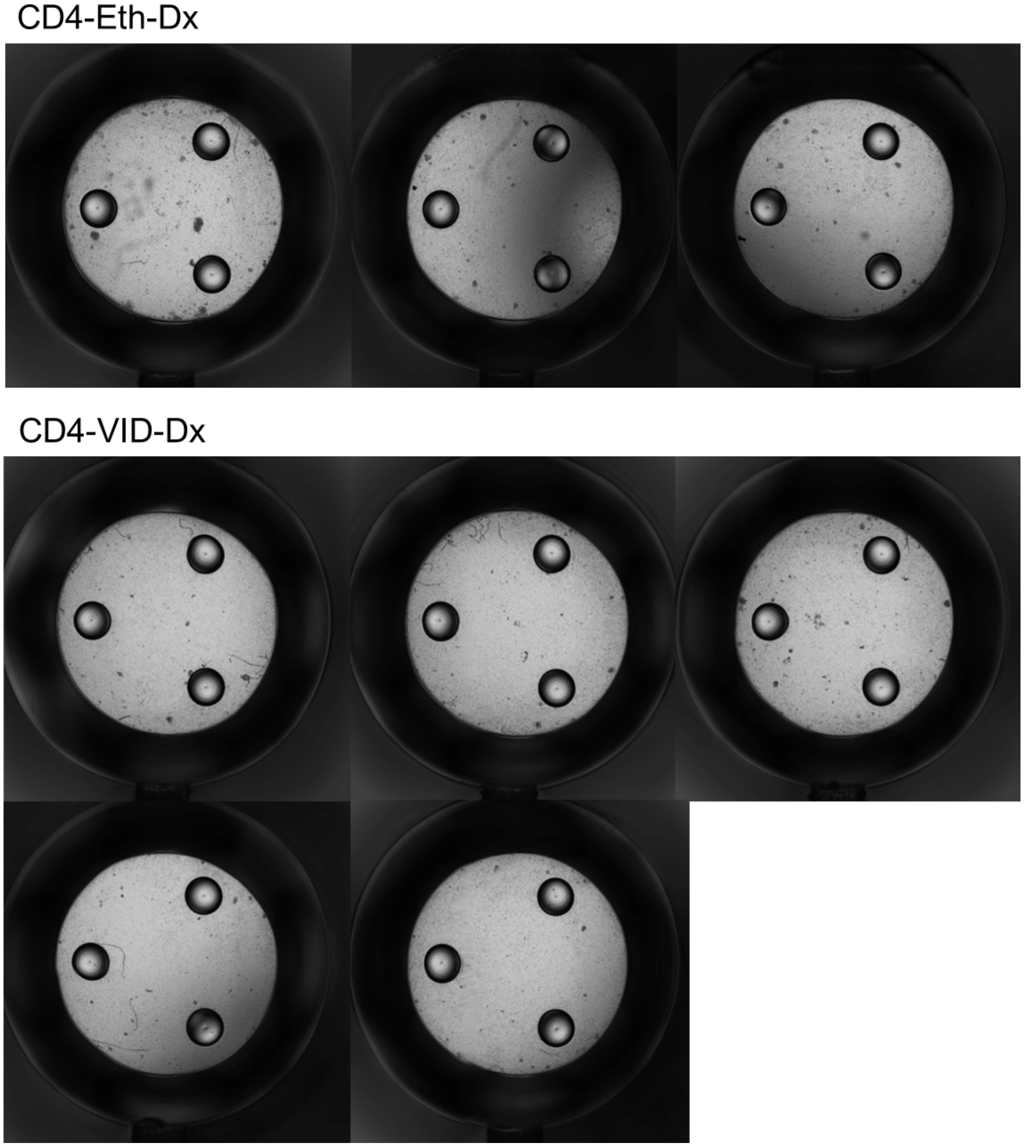

Supplementary Figure 3: Image-based quality control for mitochondrial respiration determination. Brightfield images acquired using the Cytation 5 system of $CD4^{+}$ T cell wells carrier (CD4-Eth-Dx) and VitD (CD4-VID-Dx), selected for mitochondrial oxygen consumption analysis. All wells exhibited a uniform monolayer distribution (image) and appropriate responsiveness to electron transport chain (ETC) modulators.

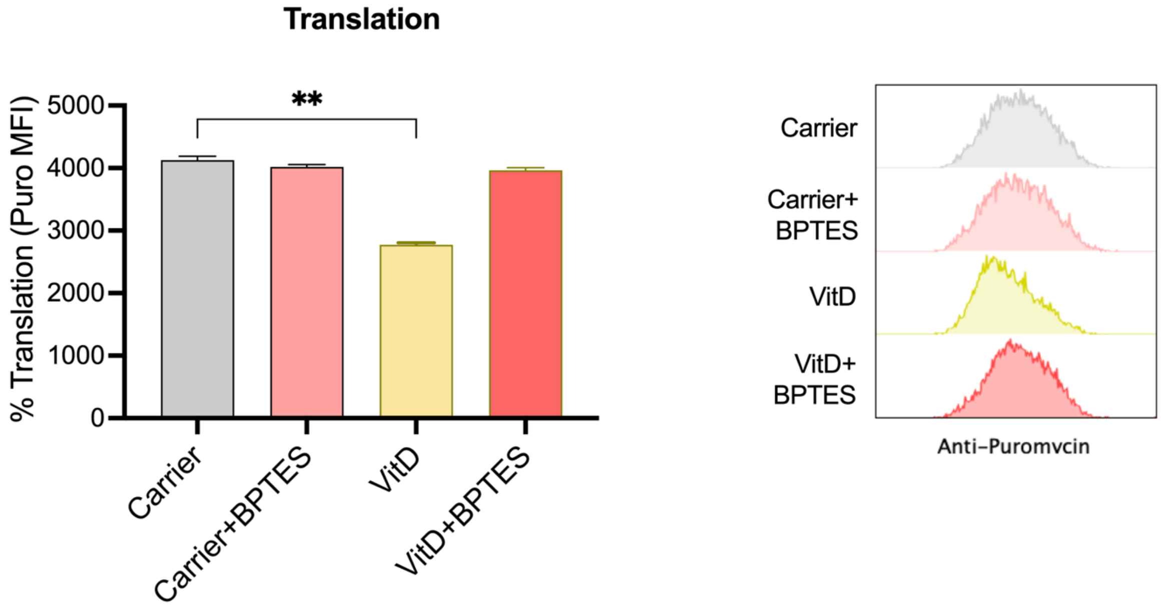

Supplementary Figure 4: Protein synthesis in $CD4^+$ T cells in the presence or absence of VitD and BPTES. Protein translation levels by puromycin MFI between $CD4^+$ T treated and untreated with VitD, N=4.
